# Informing Wildlife Corridor Creation through Population Genetics of an Arboreal Marsupial in a Fragmented Landscape

**DOI:** 10.3390/genes14020349

**Published:** 2023-01-29

**Authors:** Ana Gracanin, Monica L. Knipler, Katarina M. Mikac

**Affiliations:** Centre for Sustainable Ecosystem Solutions, School of Earth, Atmospheric and Life Sciences, Faculty of Science, Medicine and Health, University of Wollongong Australia, Wollongong 2522, Australia

**Keywords:** barriers, DArTseq, fragmentation, arboreal, mammal, tree-dwelling, connectivity, corridor

## Abstract

Habitat loss and fragmentation contribute significantly to the decline of arboreal mammal populations. As populations become fragmented and isolated, a reduction in gene flow can result in a loss of genetic diversity and have an overall impact upon long-term persistence. Creating wildlife corridors can mitigate such effects by increasing the movement and dispersal of animals, thus acting to reduce population isolation. To evaluate the success of a corridor, a before–after experimental research framework can be used. Here, we report the genetic diversity and structure of sugar glider (*Petaurus breviceps*) sampling locations within a fragmented landscape prior to the implementation of a wildlife corridor. This study used 5999 genome-wide SNPs from 94 sugar gliders caught from 8 locations in a fragmented landscape in south-eastern New South Wales, Australia. Overall genetic structure was limited, and gene flow was detected across the landscape. Our findings indicate that the study area contains one large population. A major highway dissecting the landscape did not act as a significant barrier to dispersal, though this may be because of its relatively new presence in the landscape (completed in 2018). Future studies may yet indicate its long-term impact as a barrier to gene flow. Future work should aim to repeat the methods of this study to examine the medium-to-long-term impacts of the wildlife corridor on sugar gliders, as well as examine the genetic structure of other native, specialist species in the landscape.

## 1. Introduction

Habitat loss and fragmentation are the greatest threatening processes for mammalian species in the Anthropocene [1]. Globally, 27% of mammal species are threatened with extinction because of habitat loss and decline in quality [2]. Habitat fragmentation is the reduction in continuous habitat into smaller, disjunct patches within a dissimilar matrix [3,4]. Remnant fragments are often too small and isolated to maintain viable populations of some species, and through environmental, demographic, and genetic changes, a vortex of extinction can form [5,6,7].

The effects of habitat fragmentation are particularly pronounced for arboreal species; those that are strictly bound to the canopy. Mammals that are obligate arboreal, with limited dispersal capability in a matrix that is cleared, are some of the most susceptible to extinction [8]. The distribution and abundance of an arboreal species can be reduced within a fragmented landscape as movement and connectivity within the landscape are restricted when the matrix contains no trees [9,10]. In some cases, reductions in body size have occurred due to the limited amount of habitat and resources available [11].

Fragmentation can affect the population genetic diversity and structure within arboreal mammal populations due to the isolation of patches and the inability for individuals to disperse [12,13,14,15,16]. This reduction in connectivity can lead to a loss of allelic richness and heterozygosity and enhance inbreeding [13,17,18]. Where habitat patches lead to small, isolated populations, these are highly susceptible to bottlenecks and the effects of genetic drift [13,18,19]. In turn, this can increase the risk of localised extinction [15,20].

Wildlife corridor implementation is considered an important strategy to reconnect habitat patches in fragmented landscapes [21,22,23]. In the context of arboreal species, there are examples of where corridors are vital for arboreal mammal conservation by reconnecting isolated populations and improving gene flow [24,25,26]. Planning and evaluating these corridors that are informed by data from genetic techniques are highly valuable processes [25,27,28]. Genetic research can identify and quantify the impact that habitat fragmentation is having on populations within the focal landscape [13,15,29]. Additionally, by incorporating spatial data, landscape genetics can be used to pinpoint barriers to gene flow at the landscape scale [16,30,31,32]. The combined use of spatial and genetic techniques can be used for the identification of corridor pathways and planning, which is of great importance for ongoing species- and ecosystem-level conservation and monitoring [33].

To evaluate the implementation of these corridor conservation strategies, a before–after control–impact (BACI) design can be used [34,35]. A BACI design allows researchers to infer the impact of human disturbance by comparing control sites (undisturbed) with impacted sites (disturbed) and including data collected before and after the human disturbance for both sites. A BACI approach is a powerful tool for addressing wildlife management problems at large spatial scales. However, obtaining data from ‘control’ and ‘impact’ sites is often not feasible; alternatively, Balkenhol and Waits [34] recommend before–after (BA) or control–impact (CI) studies for addressing the impacts of barriers in landscapes.

Here, we use a BA design on the arboreal sugar glider (*Petaurus breviceps*) to assess the impact of habitat fragmentation and other landscape-scale movement barriers (i.e., highways, roads, and farmlands) prior to the establishment of a wildlife corridor in south-eastern Australia. Sugar gliders are arboreal and rely on trees for movement; however, they can glide up to 30 m to move between trees and across gaps in canopy [36,37,38]. However, despite this adaptation, the species can still be impacted by barriers, such as roads [39], farmland [40], and urbanisation [37]. Other studies have found that the species is quite opportunistic and adaptable, with populations persisting in urban and agricultural areas [36,37,39,41,42], exhibiting a wide dietary breadth [43,44] and the capacity for moving on the ground [45]. Given these varying impacts of movement barriers on sugar gliders and the potential behavioural adaptations that populations may exhibit in disturbed habitat, the impacts of habitat fragmentation may be quite apparent or largely weak. Thus, in the context of creating a wildlife corridor, our aim was to identify this impact by quantifying genetic diversity and structure and identify any existing barriers to dispersal for the sugar glider. Through this, corridor pathways could be identified as part of informing the implementation of a landscape-scale wildlife corridor that forms part of the Greater Eastern Ranges Initiative [46]. The baseline genomic data that we report for sugar gliders can be used as a comparison from which a future assessment of the success of the corridor can be measured.

## 2. Methods Section

### 2.1. Study Area

**Figure 1:**
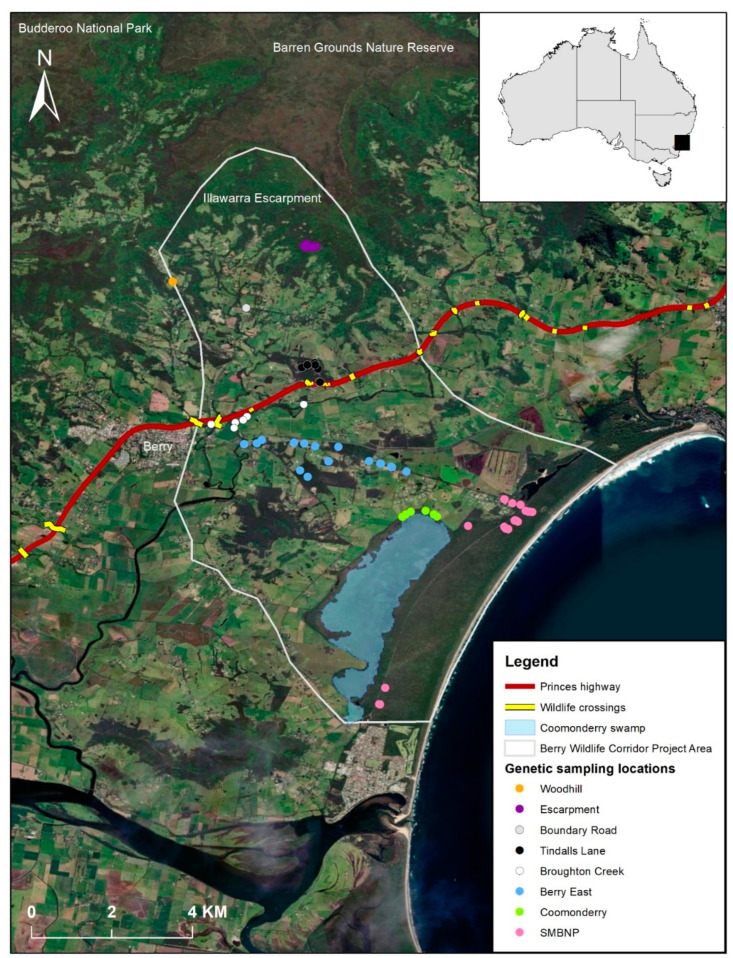
Location of where sugar glider (*Petaurus breviceps*) genetic samples (n = 94) were obtained from eight locations in the fragmented landscape surrounding the town of Berry, NSW, Australia. In the legend, SMBNP refers to Seven Mile Beach National Park. Upgrades to the Princes Highway were completed in 2018.

This study occurred in a fragmented landscape near the township of Berry (34°46′32.8″ S 150°41′56.0″ E), located approximately 100 km south of Sydney, Australia (Figure 1). The area is part of the Berry Wildlife Corridor [46], and our study captures the before data as part of a BA design. This before data will aid in evaluating the implementation of the landscape corridor involving: (1) restoring through planting and creating new habitat corridors; (2) rehabilitating existing habitat by improving its quality (hollow supplementation, weed removal, and pest removal); and (3) creating an artificial road crossing (underpasses and rope bridges over a highway (Princes Highway). The goal of the Berry Wildlife Corridor is to reconnect the isolated Seven Mile Beach National Park (SMBNP), with contiguous landscape (i.e., Illawarra Escarpment, including Budderoo National Park and Barren Grounds Nature Reserve) (Figure 1). Clearing of habitat in this region began in the early 1800’s, with extensive clearing for farmland occurring by the 1850’s, indicating that SMBNP was isolated for approximately 200 years [47].

### 2.2. Live Trapping and Tissue Collection

Live trapping occurred across 25 sites between 2018 and 2020, and 94 sugar glider individuals were caught at 16 of these sites. Based on proximity and the surrounding matrix surrounding these sites, these samples were pooled into eight groups for analyses (Figure 1). The eight locations were assigned based on apparent isolation observed from available habitat, habitat type, and barriers observed in the field (e.g., roads, trainlines, farms). Additional movement data of sugar gliders in the study area also informed the eight locations based on known spread and overlap of individual movements between areas [48]. Sugar gliders were caught using Elliott A traps [49] secured to platforms and positioned at heights of 2 m. The entrance of the trap faced the tree, and a bait of peanut butter, honey, and oats was used [39,50]. A mixture of honey water was sprayed up and down the tree as an additional olfactory attractant [51,52]. A metal ear punch (2 mm) was used to sample ear tissue from individual gliders [53,54]. The ear punch was sprayed with 70% ethanol and flamed for sterilization prior to and after ear tissue were sampled. Each tissue punch was stored in sterilized vials containing 80–95% ethanol. The DNA samples were kept at −20 °C prior to DNA extraction.

### 2.3. SNP Genotyping and Filtering

A total of 94 sugar glider tissue samples were sent to Diversity Arrays Technology Pty Ltd. (DArT) (Australia) for DNA extraction followed by next-generation sequencing. Through next-generation sequencing and genome-complexity-reduction methods, DArTseq is able to use bi-allelic, codominant SNP markers [55]. The resulting DNA fragments were then amplified with PCR and sequenced on Illumina Hiseq2500. Reads were cleaned, barcodes were removed, and sequences were aligned to the closest available relative for which a genome is available: the Leadbeater’s possum (*Gymnobelideus leadbeateri*, GCA_011680675.1 LBP_v1′) using DArTseq analytical pipelines. Quality control filtering ensured high average read depth per locus (20 reads per locus average across all markers). For additional information regarding the DArTseq process and associated quality control procedures, refer to [55].

Once the DArTseq filtering was complete, 24,682 genome-wide SNPs were retained from high-quality samples with a minimum sequence identity of 70%. Further filtering was conducted in R 4.0.2 [56] using the dartR package [57]. SNPs were filtered on call rate (0.95 threshold), reproducibility (0.99 threshold), and read depth (<5, >100), and secondaries were removed as they were likely linked. Potential loci under selection were identified (n = 13, genomic inflation factor = 1.2) and were removed using the ‘pcadapt’ package and a Bonferroni correction (*p* < 0.05) [58]. No monomorphic loci remained in the dataset. A total of 5999 SNPs were retained from 94 sugar glider individuals from eight putative populations spanning 12 km of the landscape in the sampling locations (Figure 1).

### 2.4. Genetic Diversity Analyses

All analyses were undertaken in R 4.0.2 [56]. For locations with >2 individuals, the average observed heterozygosity (H_o_) and expected heterozygosity were calculated at each SNP (H_e_) using the package ‘hierfstat’ [59]. These values were used to calculate both overall genetic diversity and coefficients of inbreeding (F_IS_) for all gliders across the entire landscape, as well as for each putative population using all 5999 genome-wide SNPs.

### 2.5. Population Genetic Structure Analyses

To examine population genetic structure, pairwise F_ST_ values were calculated between the eight sampling locations using the ‘dartR’ R package [57] with 10,000 permutations, and *p*-values were adjusted with a Bonferroni correction (see Results for details) [60]. An analysis of molecular variance (AMOVA) was calculated in the ‘ade4’ R package [61]. A principal coordinates analysis (PCoA) was performed using the ‘dartR’ package to visualise genetic distances between individuals and sampling locations [62]. Lastly, using the ‘adegenet’ R package [63], discriminant analysis of principal components (DAPC) analysis was performed to identify the most probable number of clusters (*K*) contributing to differentiation between sampling locations [64]. The *K* value selected was based on the lowest value of Bayesian information criterion (BIC) values, and where the following *K* values changed by negligible amounts [64]. As all analyses conducted indicated one large population (*K* = 1), further STRUCTURE analyses were not warranted, as bias within the analyses would lead to detection of artificial genetic clusters [65].

### 2.6. Isolation by Distance Analyses

We tested if fragmentation of habitat would lead to a decrease in landscape functional connectivity by impeding gene flow among sampling locations. This was performed by comparing two alternative models to predict patterns of genetic structure. Firstly, a null model, the isolation-by-distance (IBD) model, assumes that gene flow is only constrained by geographic distance and that genetic differentiation between sampling locations is expected to increase with Euclidian distance [66]. The second model, a landscape connectivity model, accounts for habitat heterogeneity and its potential in enhancing or impeding gene flow by relying on ecological distances. This second model used a least-cost path (LCP) analysis, using a friction layer to account for habitat suitability and barriers (values ranged from least friction of 0 to most friction with a value of 1). This was achieved by inverting a habitat suitability model created through Maxent using elevation, aspect, slope, soil type, and vegetation type to inform the model [67]. Correlating landscape distances (either Euclidean distances or LCP distances) with genetic distances enabled an understanding of whether genetic distance was affected by distance alone, or if fragmentation and barriers better explained the differentiation [68].

## 3. Results

### 3.1. Genetic Diversity

Observed heterozygosity was lower than expected for all sampling locations, except for the Woodhill location (Table 1). The F_IS_ values were greater than zero, indicating some amount of inbreeding, whereas for the Woodhill location there was a negative value, indicating an avoidance of inbreeding (Table 1).

Across the entire study area, all pooled individuals had a low F_ST_ value of 0.045 and a low F_IS_ value of 0.041. For all individuals, an overall value of 0.195 was calculated for observed heterozygosity, and expected heterozygosity was 0.203.

### 3.2. Population Structure

**Figure 2:**
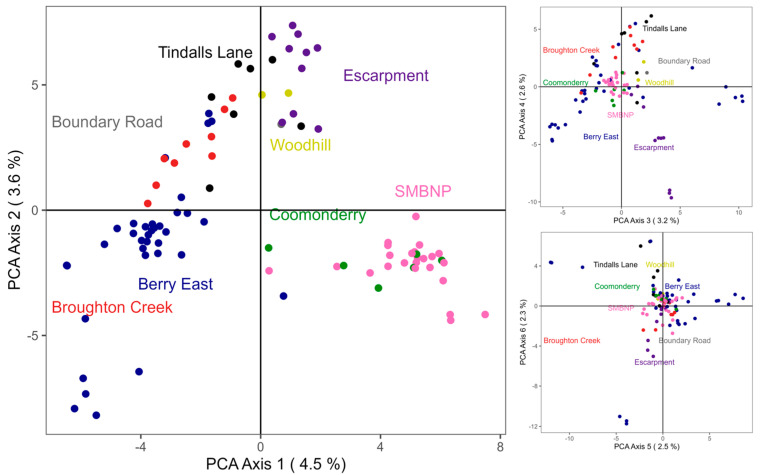
Principal coordinates analysis (PCoA) based on the genetic distances of 94 sugar glider individuals from 8 sampling locations using 5999 SNPs. Dots represent each individual. Colours represent their respective sampling location. (**Left**): representation of the first and second PCoA axes, followed by the third and fourth (**top right**) and fifth and sixth (**bottom right**).

Differentiation between individuals and sampling locations was observed (Figure 2); however, the percentage of variation explained by the first two axes was low (Figure 2). The first six axes explained a total of 18.7% of the variation, and axis 1 and 2 only explained 4.5% and 3.6%, respectively. Along PCA axis 1, individuals from SMBNP and Coomonderry clustered together and separated out from the other locations (Figure 2). Along the second axis, a mostly geographical trend of locations from generally eastern areas clustered, before increasing towards more western locations (Escarpment). Similarly, the more western locations of the Escarpment appeared to separate further from the other locations along the fourth axis. However, most individuals and locations had a high degree of overlap across axes three to six (Figure 2).

The AMOVA found that most of the genetic variation (90.03%) was explained by within-individual sugar glider variations, while only 5.12% of the genetic variation could be attributed to individuals within sampling locations (Table 2). The remaining 4.86% of genetic variation was explained by sampling locations (Table 2).

Pairwise genetic population structure estimates were all significant except for the comparison between the locations of Boundary Road and Tindalls Lane (Table 3). Pairwise F_ST_ values that were found to be significant included the adjacent SMBNP and Coomonderry (F_ST_ = 0.011), which had low genetic differentiation, whereas higher genetic differentiation (F_ST_ = 0.090) was found between Woodhill (located west of the highway) and Coomonderry, located east of the highway, 7.51 km away (direct line of distance).

**Figure 3:**
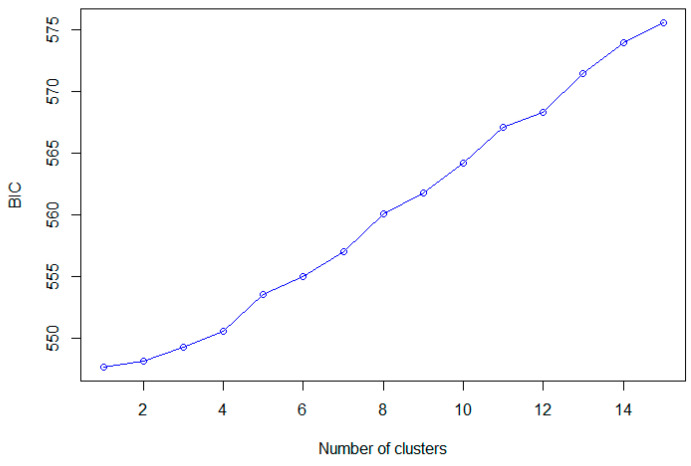
Bayesian inference criterion (BIC) values versus numbers of clusters (K), ranging from 1 to 15. Results suggest that K = 1 was the most likely number of genetically distinct clusters.

The DAPC analysis found BIC values to increase with the number of clusters tested (Figure 3). There was no deviation from this pattern as BIC maintained a steady increase. As the lowest BIC value returned K = 1, no further DAPC analyses were undertaken, as one overall population was identified.

### 3.3. Isolation by Distance

**Figure 4:**
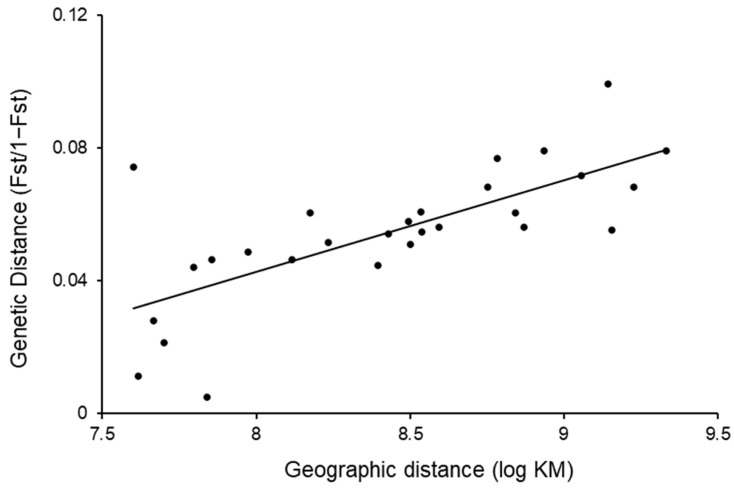
Relationship between genetic distances (F_ST_/(1 − F_ST_)) and logarithm of geographic distances (km) for eight locations of sugar gliders in the Berry Wildlife Corridor, Berry NSW.

**Figure 5:**
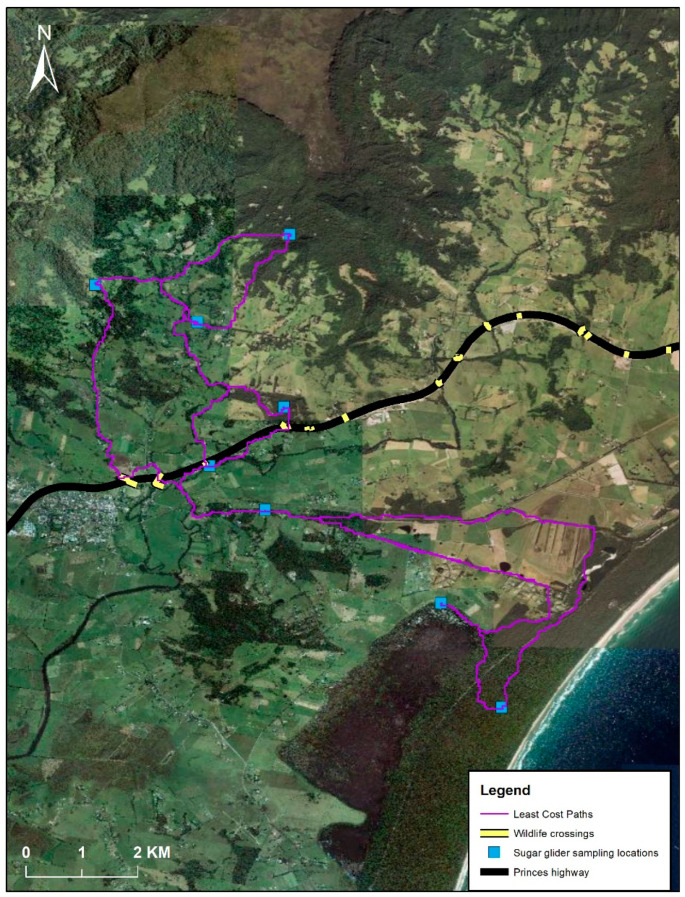
Least-cost paths tested among eight sampling locations within the Berry Wildlife Corridor area.

The IBD analysis found a strong relationship between genetic and geographic distance (Mantel’s r = 71.85%, *p* = 0.0004) (Figure 4). A least-cost path (LCP) analysis found no significant relationship between the geographic distance (least-cost path distances) and genetic distance (Mantel’s r = 35.90%, *p* = 0.0754). The LCP analysis identified several wildlife corridor pathways of connected canopy and routes that utilised wildlife crossings over the highway (Figure 5).

## 4. Discussion

The gene flow of arboreal marsupials can be negatively affected by fragmentation. This is particularly relevant where intense agriculture [14,40,69], urbanisation [16], and highways [39] act as barriers, limiting the number of individuals that can disperse long distances and enhance gene flow. In this study, we investigated how fragmentation affected gene flow to provide baseline population genetic data on a key local species, the sugar glider, before the implementation of a wildlife corridor. We found that the impacts of limited habitat connectivity within the agricultural landscape did not cause significant reductions in genetic diversity or significantly influence genetic structure in sugar gliders. Instead, we found that the entire landscape could be considered as one population for this species. Overall, the sugar gliders in the landscape studied had low pairwise population genetic structure, low levels of inbreeding, and high levels of genetic diversity.

No population genetic structure was observed between the sampling locations within the study landscape. Results from the PCoA, AMOVA, DAPC, and pairwise F_ST_ analyses all indicated one large population. A strong IBD effect was found for our null model, where geographic distance between sampling locations correlated strongly with genetic distances. This indicates that the landscape does not contain significant barriers to movement.

For the sugar glider, one study found that genetic structure and diversity (calculated using microsatellite methods) was impacted by habitat fragmentation [40]. Their study area however spanned over 40 km and contained highly isolated fragments, thus having a substantial impact on sugar glider gene flow. In comparison, our study occurred at a much finer, though still landscape scale (spanning over 12 km). Our study area also contained thin strips of roadside habitat and the presence of large and old (>200 years of age) paddock trees, which likely contributed to connectivity between sugar gliders within the landscape [36,43]. Only one other study utilised genome-wide SNP markers to investigate the effect of habitat fragmentation on sugar glider population genetics [39]. They found sugar gliders in a fragmented landscape had high levels of gene flow and little genetic differentiation, despite the biogeographical barrier of a large lake, highly urbanised towns and suburbs, and the presence of a large motorway. The study estimated that only 5.4% of sugar glider genetic variation was explained by geographic location, and there was evidence for only two genetic clusters. Similarly, only 4.86% of variation was explained by location in our study, and no structure was detected. Thus, the sensitivity of sugar gliders to habitat fragmentation is likely dependent on the pattern and quality of habitat connectivity [39,40].

Despite the limited genetic structure found in our study, there is evidence of genetic structure in fragmented populations in many other species similar to the sugar glider. For several populations of the squirrel glider (*Petaurus norflocensis*), evidence of a genetic structure has been found, which was attributed to the presence of barriers and habitat fragmentation [16,70,71]. Other arboreal mammal species, because of fragmentation, have experienced reductions in genetic diversity and increases in genetic structure. This includes species such as the common ringtail possum (*Pseudocheirus peregrinus*) [14], western ringtail possum (*Pseudocheirus occidentalis*) [72], edible dormouse [73], hazel dormouse (*Muscardinus avellanarius*) [74], and black and gold howler (*Alouatta caraya*) [75]. These arboreal mammals have adaptations for climbing or gliding between trees, and when forced to cross between suitable habitat on the ground, this increases the risk of predation and vehicle collision. Generally, such species will avoid moving through the matrix; however, for the sugar glider, the species has some resistance to disturbance. Despite being specialised for an arboreal lifestyle, sugar gliders are also highly adaptable to change and able to compete in a poorer habitat [45,48,76,77,78]. Additionally, we have shown in our other work that sugar gliders are highly mobile and able to move on the ground [45]. Thus, the species is likely be able to cross unsuitable habitat to access more suitable habitat, either by gliding (maximum glide distance of 30 m; [38]) or crossing the ground if needed.

Our study was conducted in the context of a wildlife corridor program. The program aims to revegetate missing habitat links and rehabilitate existing patches of habitat for several native ground-dwelling and arboreal species [46]. Previous research within this wildlife corridor project area was performed on the ground-dwelling brown antechinus (*Antechinus stuartii*) [79]. The study used the mtDNA cytochrome b gene and found that although sampled sites across the landscape were quite diverse, genetic structure and limited gene flow were detected for several locations within the Berry Wildlife Corridor project area. Another study within the Berry Wildlife Corridor project area identified corridor pathways for eight arboreal mammal species, which was performed using spatial analyses and ecological data through Maxent modelling [67]. It was found that connectivity existed; however, further action was required to overcome pinch-points in connectivity. In particular, the major highway presented a significant barrier, and there were minimal detections noted of arboreal mammals using the rope bridges and underpasses to cross into the habitat either side of the highway [67]. The highway is up to 80 m wide following its recent upgrade completed in 2018, which surpasses the gliding threshold for the sugar glider (30 m glide distance). Furthermore, within the corridor project area, an endangered population of greater gliders (*Petauroides volans*) is threatened by isolation at SMBNP. The species is absent from the fragmented landscape between SMBNP (located coastally) and the continuous habitat of the Illawarra Escarpment (located West; see Figure 1). Recent genetic work has found the greater gliders at SMBNP have more inbreeding than other populations and are differentiated from other populations, due to approximately 200 years of isolation following habitat loss and fragmentation [80]. Although the population genetics of sugar gliders in this study were not impacted by fragmentation, other arboreal mammal species within this corridor project area are at risk of further decline.

Sugar gliders can still be used to evaluate the effectiveness of the wildlife corridor in the long term [25,34]. Changes in genetic structure and diversity may reflect positive changes in response to improved habitat connectivity and an increase in available habitat. However, if ongoing development and habitat loss destroy important linkages of habitat then we may detect negative changes in their population genetics. Furthermore, the impact of the recent highway upgrade may have significant long-term consequences on gene flow for the species on either side of the highway.

## 5. Conclusions

The impacts of the wildlife corridor are likely to take effect between now and the next 100 years, as planted trees reach maturity and begin to form the critical hollows needed by arboreal mammals for denning and raising young [81]. We provided a baseline of genetic data for the sugar glider, which is found widespread across the landscape, to evaluate the impact of recreating new habitat links and rehabilitating existing habitat. Currently, sugar gliders within the study landscape have high levels of genetic diversity and gene flow and limited genetic structure. This further exemplifies that the species can make use of low-quality habitat with limited connectivity. However, future threats in the landscape may impact this species (recent highway upgrade, development and habitat clearing) and therefore a repetition of this study is needed in the future.

## Figures and Tables

**Table 1:** Observed heterozygosity (H_o_), expected heterozygosity (H_e_), and inbreeding coefficient (F_IS_) for 94 sugar glider individuals across eight sampling locations within the Berry Wildlife Corridor, Berry NSW, Australia. Total sample size of 94 individuals and calculations used 5999 SNPs in total. Standard deviation is shown in parentheses.

Sampling Location	N	Ho	He	F_IS_
Berry East	35	0.188 (0.182)	0.200 (0.184)	0.045 (0.203)
Boundary Road	1	0.212 (0.409)	-	-
Broughton	9	0.190 (0.206)	0.202 (0.197)	0.042 (0.304)
Coomonderry	6	0.189 (0.228)	0.195 (0.207)	0.009 (0.352)
Escarpment	10	0.202 (0.210)	0.208 (0.194)	0.021 (0.297)
SMBNP	24	0.189 (0.185)	0.202 (0.185)	0.053 (0.228)
Tindalls Lane	7	0.187 (0.209)	0.207 (0.205)	0.069 (0.341)
Woodhill	2	0.202 (0.314)	0.196 (0.275)	−0.126 (0.534)

**Table 2:** Percentages of molecular variance within sugar glider individuals, between individuals within sampling locations, and between sampling locations.

Source of Variation	D.F.	SS	MS	% Variation	*p*
Between locations	7	8905.74	1272.249	4.86	0.001
Between individuals within sampling locations	86	54,804.95	637.267	5.12	0.001
Within individuals	94	53,790.56	572.240	90.03	0.001
Total	187	117,501.25	628.349	100	

**Table 3:** Pairwise population F_ST_ values between sugar glider sampling locations (below the diagonal), and significant comparisons were Bonferroni corrected. *: *p* < 0.0017; n.s.: *p* > 0.0017.

	SMBNP	Broughton Creek	Boundary Road	Berry East	Woodhill	Escarpment	Coomonderry	Tindalls Lane
SMBNP	-	*	*	*	*	*	*	*
Broughton Creek	0.053	-	*	*	*	*	*	*
Boundary Road	0.052	0.044	-	*	*	*	*	n.s.
Berry East	0.048	0.021	0.055	-	*	*	*	*
Woodhill	0.073	0.051	0.069	0.072	-	*	*	*
Escarpment	0.064	0.053	0.042	0.064	0.057	-	*	*
Coomonderry	0.011	0.052	0.073	0.046	0.090	0.067	-	*
Tindalls Lane	0.057	0.027	0.005	0.044	0.043	0.049	0.057	-

## Data Availability

DArTseq data is available online through the figshare digital repository: https://figshare.com/articles/dataset/Berry_wildlife_corridor_sugar_glider_DArTseqdata/21972329 (accessed on 5 December 2022).
